# Synergistic and Antagonistic Effects of Aromatics on the Agglomeration of Gas Hydrates

**DOI:** 10.1038/s41598-020-62060-5

**Published:** 2020-03-26

**Authors:** Tai Bui, Deepak Monteiro, Loan Vo, Alberto Striolo

**Affiliations:** 1 Department of Chemical Engineering, University College London, WC1 E7JE London, UK; 2 Halliburton, Houston, Texas USA

**Keywords:** Chemical engineering, Colloids

## Abstract

Surfactants are often used to stabilize aqueous dispersions. For example, surfactants can be used to prevent hydrate particles from forming large plugs that can clog, and sometimes rupture pipelines. Changes in oil composition, however dramatically affect the performance of said surfactants. In this work we demonstrate that aromatic compounds, dissolved in the hydrocarbon phase, can have both synergistic and antagonistic effects, depending on their molecular structure, with respect to surfactants developed to prevent hydrate agglomerations. While monocyclic aromatics such as benzene were found to disrupt the structure of surfactant films at low surfactant density, they are expelled from the interfacial film at high surfactant density. On the other hand, polycyclic aromatics, in particular pyrene, are found to induce order and stabilize the surfactant films both at low and high surfactant density. Based on our simulation results, polycyclic aromatics could behave as natural anti-agglomerants and enhance the performance of the specific surfactants considered here, while monocyclic aromatics could, in some cases, negatively affect performance. Although limited to the conditions chosen for the present simulations, the results, explained in terms of molecular features, could be valuable for better understanding synergistic and antagonistic effects relevant for stabilizing aqueous dispersions used in diverse applications, ranging from foodstuff to processing of nanomaterials and advanced manufacturing.

## Introduction

Dispersions are found in a variety of applications, from foodstuff to minerals processing, from biotechnology to nanotechnology, from 3D printing to advanced manufacturing. One relevant application in the energy sector concerns flow assurance. In oil and gas pipelines, the simultaneous presence of natural gas and water can lead to the formation of hydrate plugs, which could clog, and sometimes rupture the pipeline. In addition, gas hydrates can form in many offshore energy processes^1^. The unintended formation of hydrate plugs can cause disruptions in oil and gas production, as well as large negative environmental consequences^1–3^. Among other approaches to manage gas hydrates is the stabilization of hydrate particles in hydrocarbon dispersions. Specifically designed surfactants, known as anti-agglomerants (AAs), are optimized to prevent hydrate plug formation in flow assurance^4,5^. AAs are believed to adsorb on hydrate particles by their hydrophilic head groups, while the AAs tail groups are soluble in the hydrocarbon phase. The hydrate particles, as well as water droplets, are expected to be covered by a film of AAs and oil, making them repel each other and disperse^5–9^. This rich system offers an ideal platform to test our fundamental understanding regarding the stabilization of dispersions using surfactants. In fact, laboratory and field observations alike show that many phenomena determine the AAs’ performance. Small changes in the molecular structure of the surfactants, changes in salt content, and in gas/oil and in water/oil ratios can strongly affect the ability of surfactants to prevent hydrate plugs formation. Of particular interest is the fact that the performance of AAs also depends on the type of oil. Crude oil consists of many different components, including light ones such as alkanes, and heavy ones such as asphaltenes and resins. Some crude oils show excellent anti-agglomeration performance without AAs^10–13^. It has been suggested that polycyclic aromatic compounds can act as natural AAs^13,14^. Others report that carboxylic acid – based compounds can behave as natural AAs^11,15,16^. The chemical structure of natural AAs is unknown, as are the mechanisms of action of such compounds. Considering other production, transportation and oil treatment processes, it has been shown that the stability of water-in-oil (W/O) emulsions is dependent on the rigidity of the interfacial film, which is in some cases composed mainly of asphaltenes, resins, and fatty acids^17–21^. Different oil components, as well as additives affect the interfacial film, hence the emulsion stability. For instance, low-molecular-weight aromatics and their surface-active derivatives have been found to destabilize W/O emulsions when asphaltenes are present^22–24^. It has also been reported that, depending on resin/asphaltene ratios, the W/O emulsion stability can be enhanced or reduced by low-molecular-weight aromatics^22,25^. Understanding the mechanisms and factors responsible for the stabilization of W/O emulsion in the presence of aromatics appears to be crucial for securing technological advancements in these sectors.

In the present work, we employ classic molecular dynamics (MD) simulations to quantify how selected aromatic compounds could act as natural AAs, as well as how they might affect the performance of synthetic AAs. Five different monocyclic and polycyclic aromatic compounds were considered: benzene, toluene, p-xylene, naphthalene, and pyrene. We analyse their behaviour at the hydrate-oil interfaces, in terms of density profiles, preferential orientation, etc. As synthetic AAs, we chose a compound, recently developed, which has shown good laboratory performance in preventing hydrate formation in light oils^8^. We quantified how the AAs film at the hydrate-oil interface is affected by the presence of the aromatics. While some aromatics disrupt the AAs film, others synergistically integrate with it. The effects of aromatics on the AAs performance were quantified by estimating the free energy profiles experienced by two hydrate substrates as they approach each other. Effective repulsive interactions are assumed to positively correlate with the stability of hydrate dispersions. Those aromatics that enhance the order of AAs film were found to promote stronger hydrate-hydrate repulsions, while those that negatively affect AAs films order decreased the effective repulsion between two approaching hydrates. These results could help design AAs formulations that are effective in various crude oils, could provide important evidence to explain the performance of asphaltenes in preventing gas hydrate agglomeration, and could also help the community further the fundamental understanding of the diverse mechanisms that are responsible for the stabilization of dispersions.

## Simulation Methodology

**Figure 1:**
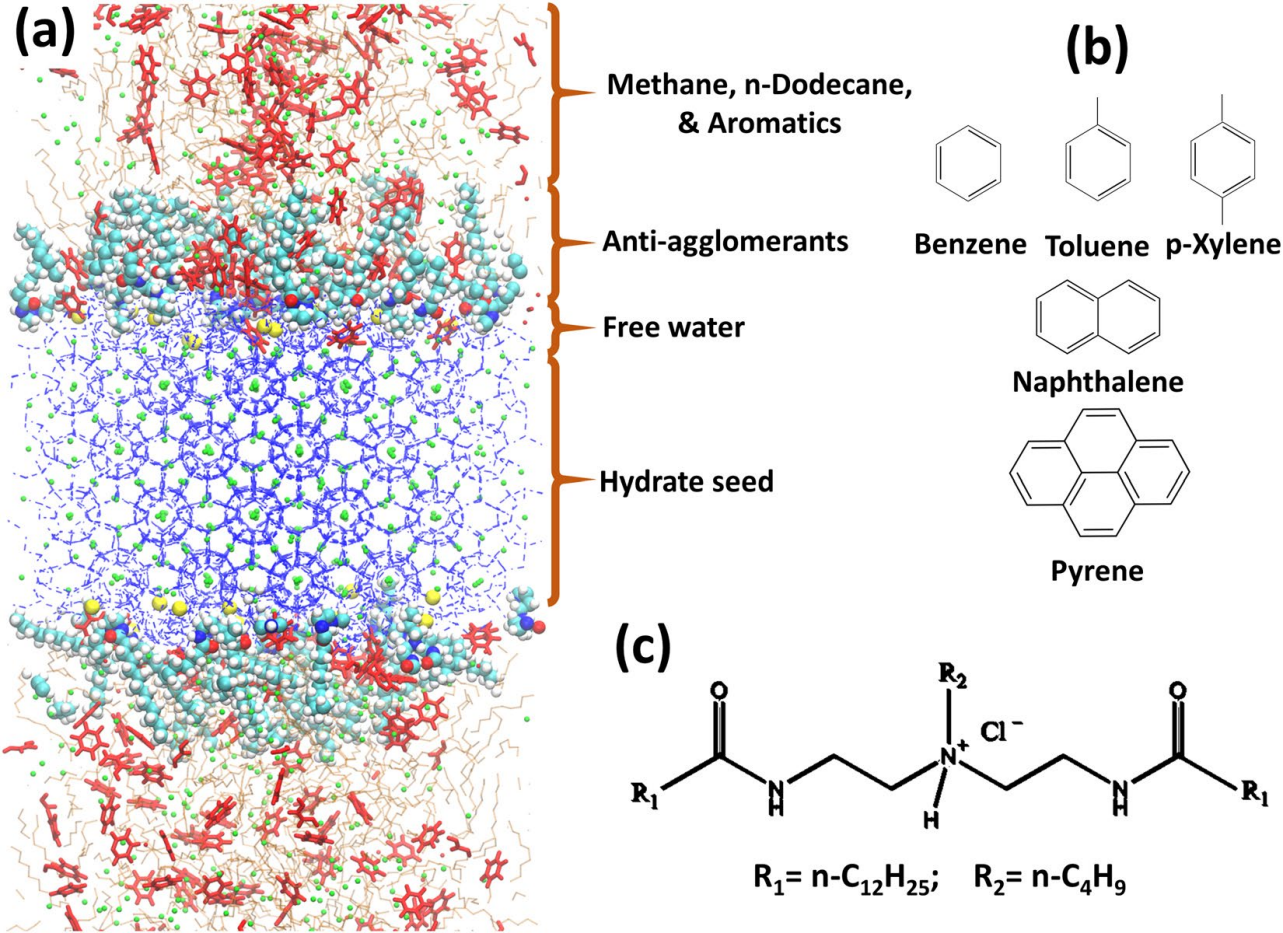
(**a**) Representative simulation snapshot. Red = aromatics, green = methane, orange = n-dodecane, blue = water, yellow = chloride ions, and cyan and white spheres = AAs. (**b**) The molecular structure of five aromatic compounds considered in this work. (**c**) The molecular structure of the AAs considered, which contain two long hydrophobic tails, R_1_, and one short hydrophobic tail, R_2_. R_1_ is composed of one n-dodecyl chain, and R_2_ of linear hydrocarbon chains of four carbon atoms.

Molecular dynamics (MD) simulations were performed using the GROMACS package^26^, version 5.1.2. One representative orthorhombic simulation box is shown in Fig. 1a. To construct the initial configurations, we followed the procedure described in our previous study^27^. Each simulated system contains a sII methane hydrate substrate of size 5.193 × 5.193 × 3.462 nm. The hydrate substrate was constructed by replicating the hydrate unit-cell adopted from Takeuchi *et al*.^28^ 3 × 3 × 2 times along X, Y, and Z directions, respectively. On top of the hydrate, along the Z direction, a thin water film was deposited (~0.5 nm in thickness) resembling the quasi-liquid layer expected on gas hydrate surfaces^29^.

Although the methane sII hydrate can be thermodynamically stable at large pressures (>100 MPa), a coexistence between sI and sII methane hydrates is generally observed at moderate pressures, as shown by experiments^30^ (similar observations hold for CO_2_ hydrates^31,32^) and simulations^33^. We confirmed that in the timescale of our MD simulations the structure of sII methane hydrate remained intact, consistent with our previous study^8^. Moreover, the underlying assumption in our simulations is that the guest molecules in the hydrates do not affect the properties of the aromatics and the AAs films adsorbed on the hydrate. This assumption is supported by the fact that the interactions between guest molecules are mainly short ranged and that on the hydrate surface there exists a ‘quasi-liquid’ layer of water^29,34,35^. The sII hydrate was chosen in the present study because it represents the hydrate structure formed in the rocking cell experiments used to test the performance of AAs^8^.

The hydrocarbon phase consists of n-dodecane, methane, and aromatic compounds. Five aromatic compounds (molecular structure shown in Fig. 1b) were simulated: benzene, toluene, p-xylene, naphthalene, and pyrene. AAs with molecular structure shown in Fig. 1c were inserted at the water-hydrocarbon interface. The compositions of the simulated systems are summarized in Table 1. In this study the mass fractions of benzene, toluene, p-xylene, naphthalene and pyrene in hydrocarbon phase are 16.0%, 18.3%, 20.6%, 23.8% and 18.8%, respectively. In crude oils the mass fraction of aromatics varies wildly, from 3% to more than 30%^36–38^. It is worth noting that the aromatics remain well dispersed in the bulk oil phase in the simulations without gas hydrates and water (temperature, pressure, and composition constant). This indicates that the concentrations of aromatics used here are within the solubility limit at the thermodynamic conditions simulated for the models implemented.

**Table 1:** Compositions of the simulated systems.

System	Number of n-dodecane molecules	Total number of methane molecules	Number of aromatics	Number of water molecules in hydrate substrate	Number of free water molecules	Number of AAs
No AAs	880	1312	400 for monocyclic aromatics and naphthalene; 200 for pyrene	2448	900	0
0.44 molecules/nm^2^ of AAs	880	1312	400 for monocyclic aromatics and naphthalene; 200 for pyrene	2448	900	12
0.89 molecules/nm^2^ of AAs	880	1312	400 for monocyclic aromatics and naphthalene; 200 for pyrene	2448	900	24

The TIP4P/Ice model^39^ was implemented to simulate water molecules. Methane and n-dodecane were represented within the united-atom version of the TraPPE-UA force field^40^. Aromatics and AAs were modeled by implementing the General Amber Force Field (GAFF), often used to simulate organic and pharmaceutical molecules containing H, C, N, O, S, P, and halogens^41^. Atomic charges in AAs and aromatics were calculated with the AM1-BCC method employed in Antechamber from the Amber 14 suite^42^. The system was maintained electrically neutral by adding chloride ions (Cl^−^), which were modeled as charged Lennard-Jones spheres with the potential parameters taken from Dang, without polarizability^43^.

A canonical ensemble simulation (NVT) was conducted for 1 ns to relax the initial configuration (the hydrate layer was kept frozen) at 277 K. Subsequently, NPT simulations were conducted at 277 K and 20 MPa. For the first 5 ns, the Berendsen thermostat and barostat were implemented to efficiently scale simulation box temperature and volumes^26^. After 5 ns, thermostat and barostat were switched to Nose-Hoover and Parrinello-Rahman, respectively^26^. The pressure coupling was only applied along the Z direction of the simulation box, which allowed us to maintain X and Y dimensions constant, and keep the same surface area for different systems. Each simulation was conducted for more than 100 ns for the systems without AAs, or at 0.44 molecules/nm^2^ of AAs in the presence of monocyclic aromatics and naphthalene; more than 200 ns were simulated for the systems at 0.89 molecules/nm^2^ of AAs, as well as for those in the presence of pyrene. Even with typical AA dosage of 0.5–2vol% in water, as gas hydrates form, grow and agglomerate, one should expect an increase in AA surface density on various interfaces, including hydrate particles, as the free water converts to hydrates and the hydrate particles increase in size. For comparison, it has been reported that simple single-tail or gemini surfactants yield surface densities as high as 2.5 molecules per nm^2^^[,44,45^. Because the AAs considered here are more complex, and certainly bulkier than single-tailed surfactants, they should yield lower surface densities. Thus, the maximum surface density considered in our simulations was of 0.89 molecules per nm^2^. The simulations were considered equilibrated when the density profiles of aromatics and the potential energy of the system converged.

## Results and Discussion

### Density profiles

The performance of surfactants in stabilizing dispersions is predicated on their ability to accumulate at solid-fluid interfaces, yielding a repulsive barrier due to steric, electrostatic, and/or dispersive interactions^46,47^. Thus, it is important to quantify the ability of the various compounds considered here to accumulate at the hydrate-hydrocarbon interface.

**Figure 2:**
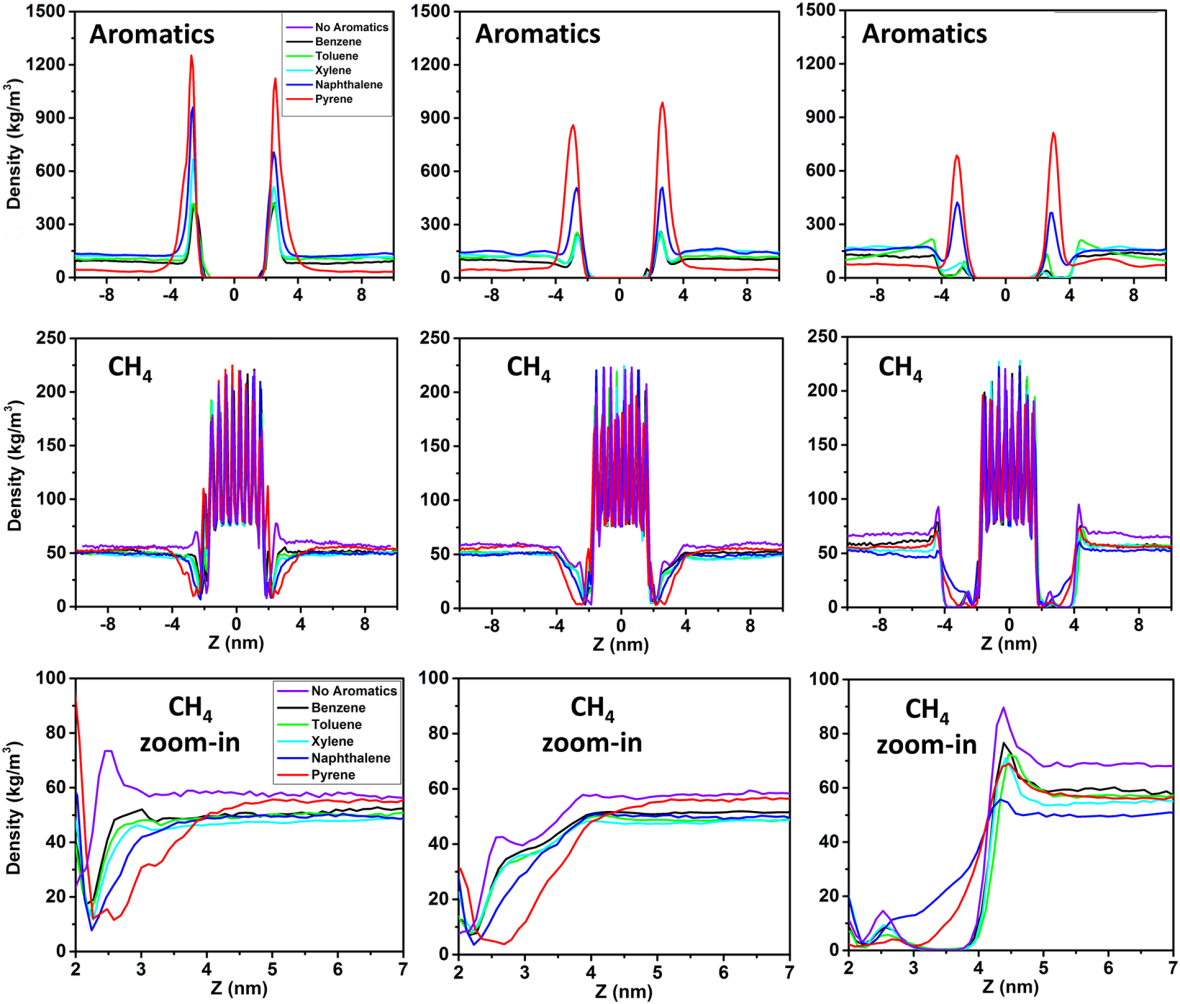
Density profiles of aromatics (top) and methane (middle), along the Z direction of the simulation box in systems without AAs (left), with 0.44 molecule/nm^2^ of AAs (middle), and with 0.89 molecules/nm^2^ of AAs (right). The zoom-in density profiles of methane near the hydrate/oil interfaces (bottom panels) were calculated by averaging the density of methane on both sides of the hydrate substrate.

In Fig. 2, from left to right, the results are shown in terms of density profiles along the Z direction of the simulation box (perpendicular to the hydrate surface) for aromatics (top panels) and methane (middle and bottom panels). Because the equilibration stage lasted approximately 50 ns, the density profiles were obtained by averaging the data collected during the last 20 ns of the simulations. The methane density profiles are shown in systems without AAs, as well as in systems with AAs at 0.44 molecules/nm^2^, and 0.89 molecules/nm^2^ (from left to right, respectively). The results show that all the aromatics considered here preferentially accumulate at hydrate-oil interfaces (Z ~ 3 nm). Some of the density profiles are not symmetric with respect to the hydrate substrate. This is because once aromatics adsorb at the interface, they hardly diffuse back to the bulk hydrocarbons within the simulation times probed here. Adsorption seems governed by the number of aromatic rings in the compounds. Polycyclic aromatics show stronger adsorption compared to monocyclic ones, as indicated by the height of the density peaks. The density of the first density peak, which represents adsorbed compounds at the interface, decreases in the order: pyrene > naphthalene > p-xylene > toluene ~ benzene.

**Figure 3:**
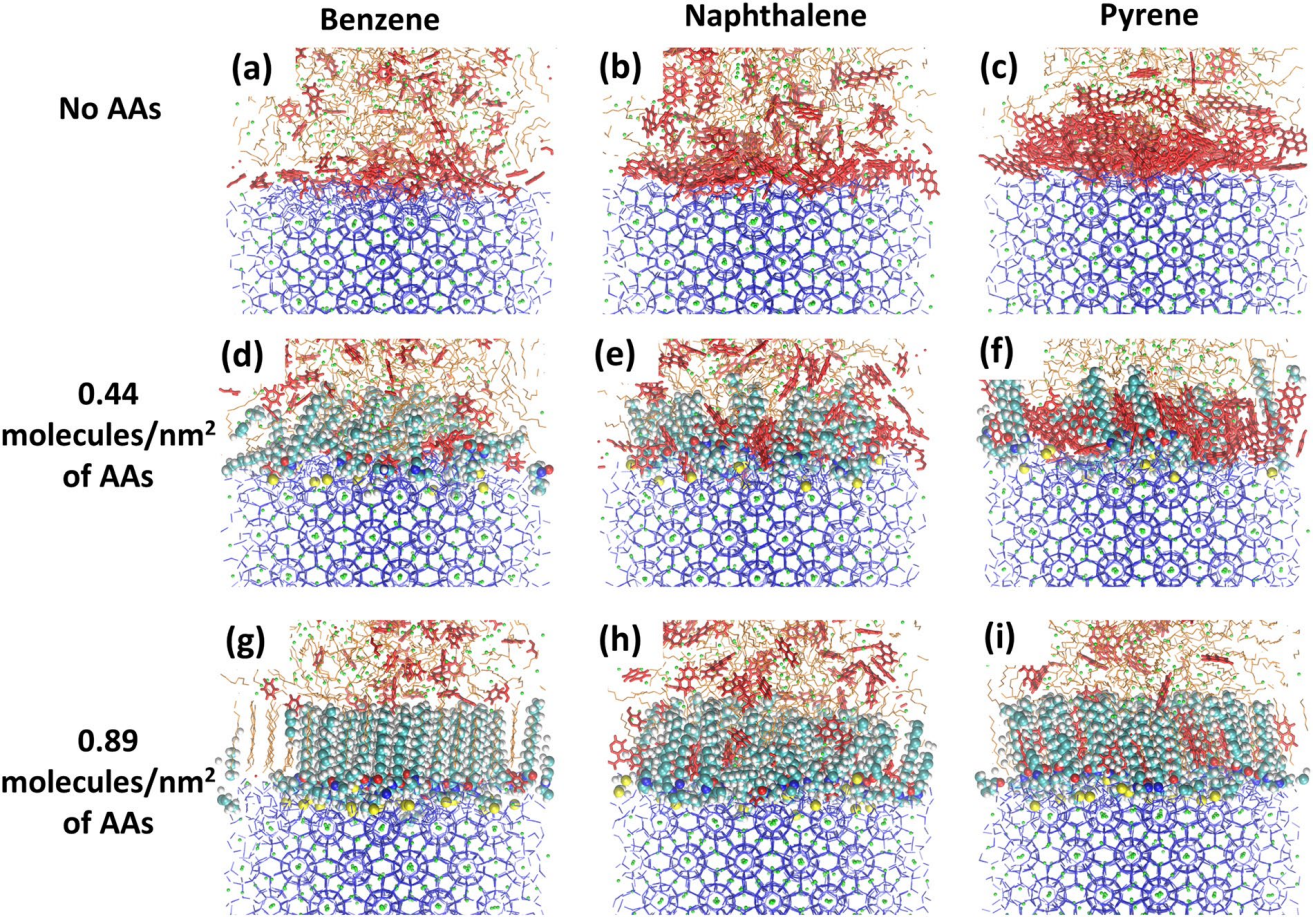
Representative simulation snapshots of the simulated systems in the presence of benzene (left), naphthalene (middle), and pyrene (right) at different surface densities of AAs: without AAs (top), 0.44 molecules/nm^2^ of AAs (middle), and 0.89 molecules/nm^2^ of AAs (bottom), respectively. The color code is described in Fig. 1.

When the AAs film is present, the density of adsorbed aromatics decreases. The results show that monocyclic aromatics are much less adsorbed than polycyclic ones. This is particularly evident when the AAs are at high surface density, at which conditions monocyclic aromatics are almost excluded from the interfacial regions (see the right panel of Fig. 2). For completeness, it should be remembered that at these high surface densities, AAs yield a well-ordered film when aromatics are not present^8^. In Fig. 3 we report simulation snapshots to illustrate the systems just discussed.

We also calculated the density profiles of methane along the Z direction of the simulation box, which is perpendicular to the hydrate substrate. The results are presented in the middle and bottom panels of Fig. 2. The presence of aromatics lowers the concentration of methane near the interfaces. Comparing the results obtained in the presence of monocyclic and polycyclic aromatics, it is found that more methane is excluded from the interfaces when polycyclic aromatics are present. This could be because polycyclic aromatics yield better packing than monocyclic ones at the interface. The lower concentration of methane at the interface in the presence of aromatic compounds could reduce the hydrate growth rate^9^, which is consistent with the experimental data obtained in the presence of asphaltenes and resins^48^. When AAs are present at low surface density, fewer methane molecules are present at the interfaces compared to results obtained when no AAs are present. This is consistent with our previous study^8^. At high AAs density (right panel of Fig. 2), the methane exclusion from the interfacial region is due to the formation of an ordered interfacial layer. Our results show that monocyclic aromatics are also excluded from the interfaces, whereas polycyclic aromatics are found within the interfacial films. Consistent with these observations, the results show that methane is excluded from the interfaces when monocyclic aromatics are present, but it can penetrate the films when polycyclic aromatics are present.

### Adsorption of aromatics at oil-hydrate interfaces

Independent bulk simulations (not reported here for brevity) confirmed that the aromatic compounds considered here were fully mixed in the hydrocarbon systems at the thermodynamic conditions tested in our simulations. Yet, the results discussed above suggest strong segregation of these compounds at the hydrate-hydrocarbon interface. What is the driving force for such strong segregation? The results above also suggest a competition between AAs and aromatics for adsorption at the interface, which depends on the size of the aromatics. What are the driving forces for this competition?

To answer the first question, we computed free energy profiles in the form of Potential of Mean Forces (PMF), and we differentiated internal energetic and entropic contributions for selected systems. We implemented the umbrella-sampling algorithm to calculate PMFs for one benzene/pyrene molecule as it adsorbs from the bulk hydrocarbon phase on the hydrate substrate. The simulations were conducted at 257 K and 277 K with one aromatic molecule and no AAs. Benzene and pyrene were allowed to oscillate around a constrained Z position, to which they were tethered by harmonic springs of elastic constant 1000 kJ/(mol.nm^2^). During umbrella sampling simulations, benzene and pyrene molecules were allowed to rotate freely. Details on the algorithm are described elsewhere^26^. In each sampling window, a production run of 10 ns was conducted in the NVT ensemble. The PMF profiles were reconstructed implementing the Weighted Histogram Analysis Method (WHAM) algorithm^49^. Error bars were estimated via the bootstrap analysis, as implemented in GROMACS^49^. From the PMFs simulated at two temperatures we extracted the entropic contribution to the free energy profile via:

1 $$\Delta S=-\,\frac{\partial \Delta F}{\partial T}\approx -\,\frac{\varDelta {F}_{\xi ,T+\Delta T}-\Delta {F}_{\xi ,T}}{\Delta T}$$

**Figure 4:**
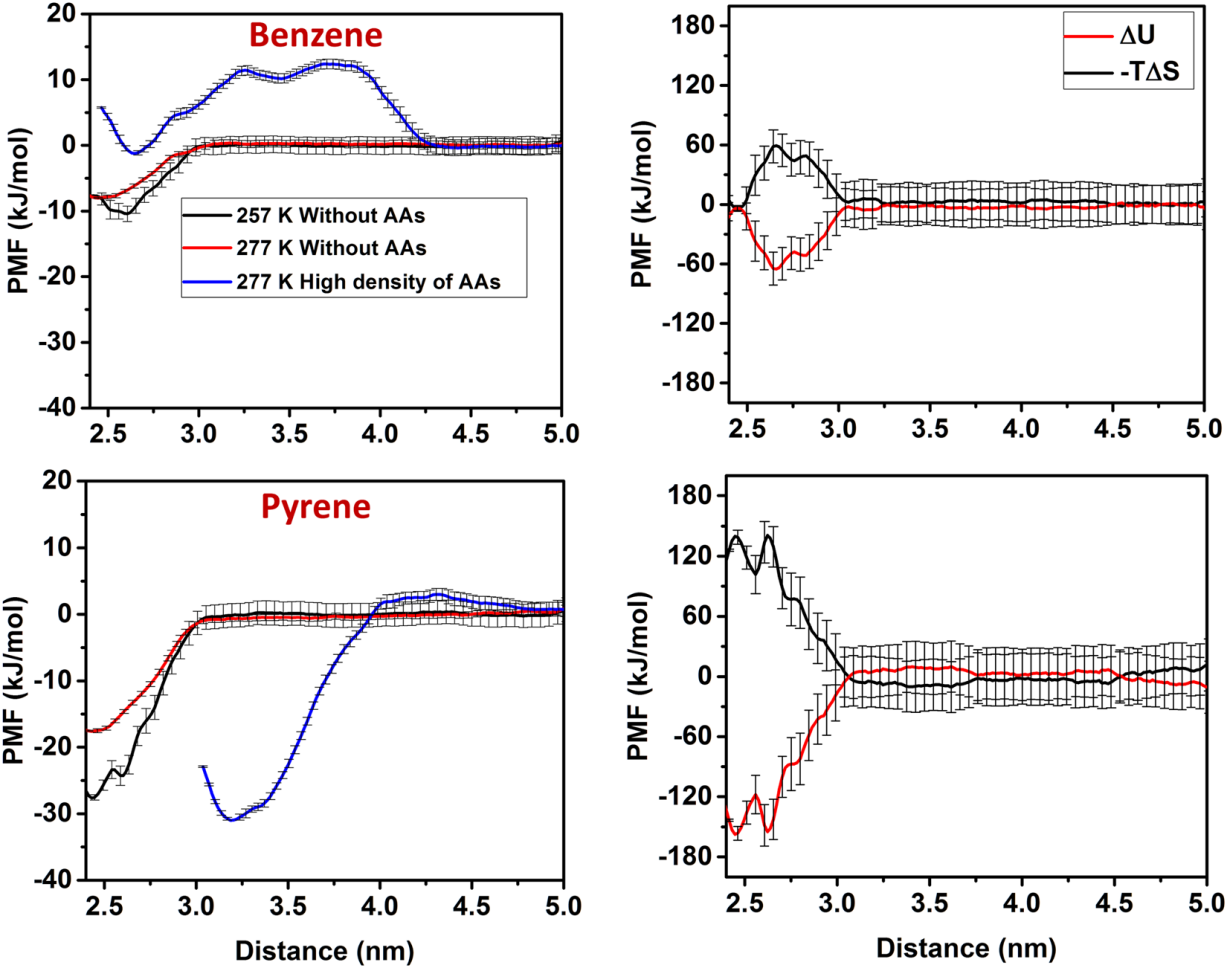
Left: PMF profiles for benzene (top) and pyrene (bottom) adsorption in the systems without AAs (black and red curves) and at high surface density of AAs (blue curves); right: entropic and internal energetic contributions for benzene (top) and pyrene (bottom) adsorption on the hydrate substrate when AAs are not present.

In Eq. (1), *F* is the Helmholtz free energy, i.e., PMF, *T* is the temperature, $\xi$ is the reaction coordinate, and $\Delta T=20K$ is the temperature difference. The internal energetic contribution to the PMFs was calculated as $\,\Delta U(\xi )=\Delta F(\xi )+T\Delta S(\xi )$. This approach has been used widely and successfully^50–52^. The results obtained are presented in Fig. 4.

The PMF profiles obtained for benzene and pyrene in the absence of AAs show no free energy barrier to adsorption, and an attractive free energy well at ~2.5 nm from the hydrate substrate. At this distance the aromatics reach the hydrate surface. A deeper free energy well is observed for pyrene than for benzene, suggesting stronger adsorption. Based on the PMF profiles, it is possible that a metastable state is present for pyrene at 257 K at ~2.6 nm, which could be due to the roughness of the hydrate surface at low temperature. The depth of the wells in the PMF profiles is consistent with the strong adsorption observed for these aromatics at the hydrate-hydrocarbon interface and discussed above (the free energy of adsorption is estimated in −8.0 ± 0.5 kJ/mol and −17.6 ± 0.5 kJ/mol for benzene and pyrene, respectively). The results obtained indicate that the adsorption of aromatics on gas hydrate surfaces is thermodynamically favourable, and that pyrene adsorbs more strongly compared to benzene, which is consistent with the density profiles shown in Fig. 2. In terms of the thermodynamics driving force, the right panels of Fig. 4 reveal that the adsorption of both benzene and pyrene is dominated by energetic contributions; in both cases, the aromatics experience an entropic penalty as they adsorb on the hydrate, penalty which is larger for pyrene than for benzene, because adsorption reduces the rotational freedom of these compounds.

Additional simulations were conducted by replacing methane in large cages of sII hydrates with propane, and by adding propane to the hydrocarbon phase at the ratio of 0.035 propane molecules per methane (which corresponds to the methane/propane ratio in Green canyon gas^8^), while keeping all other conditions un-changed. We then calculated the PMF profiles for the adsorption of one benzene and one pyrene molecule on the hydrate. The results (not shown here for brevity) are statistically indistinguishable from those presented here, within the accuracy of our simulations. This supports our assumption that the guest molecules in the hydrates do not affect the properties of aromatic - AA films on the hydrates.

The adsorption of aromatics in the presence of AAs at 0.89 molecules/nm^2^ was investigated by reconstructing the PMF profiles for one benzene/pyrene at 277 K. The final configurations of the equilibrated systems of Fig. 2 were used as the initial configurations for these simulations. The results are presented as the blue curves in the left panels of Fig. 4. In the case of benzene, a high free energy barrier is observed as the molecule moves across the AA interfacial film (from 2.65 nm to 4.5 nm). This implies that the integration of benzene into the AAs film is thermodynamically and kinetically unfavourable. Contrarily, we observe a rather low free energy barrier encountered by pyrene as it adsorbs into the AAs film. A deep free energy well at ~3.2 nm suggests that the adsorption within the AAs film is thermodynamically favourable, even more favourable than that observed when AAs are not present. This is probably due to attractive interactions between pyrene molecules and AAs. Note that the position of the free energy minimum observed for pyrene shifted to ~3.2 nm when AAs are present because the distance is measured between the center of mass of pyrene and that of the hydrate substrate. When the pyrene molecule is perpendicular to the interface (see discussion below), its center of mass shifts to larger distances.

### Orientation of aromatics at the oil/hydrate interface

**Figure 5:**
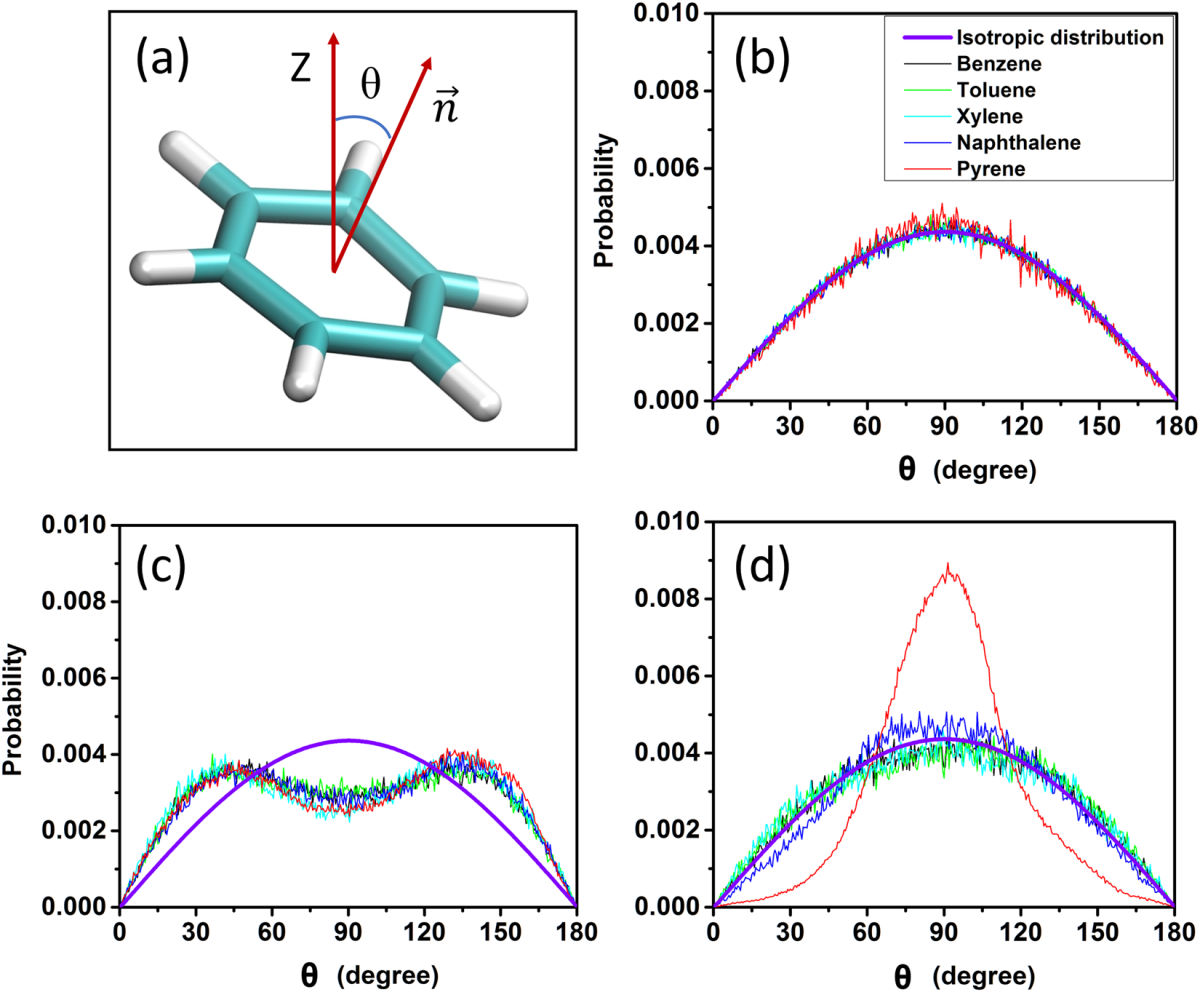
Probability distribution of the angle θ between the vector normal to the aromatic plane and the Z direction of the simulation box. In panel (**a**) a schematic of the angle θ is shown. The results are shown for aromatics in the bulk hydrocarbon phase (**b**), at the hydrate-oil interfaces without AAs (**c**), and with AAs (**d**) at a surface density of 0.44 molecules/nm^2^.

The orientation of aromatics at the interfaces is examined by calculating the probability distribution of the angle θ formed by the vector normal to the aromatics plane and the Z direction of the simulation box. The results are shown in Fig. 5, where we compare bulk vs. adsorbed aromatics. For reference, the angle distribution consistent with no orientational order is shown as a thick purple curve. In the bulk hydrocarbon phase (Fig. 5b), the angle θ follows an isotropic distribution for all aromatics. At the oil/hydrate interfaces all aromatics exhibit anisotropic orientational distributions (Fig. 5c). When AAs are not present, the angle distribution shows preference for either 0° or 180°. This means that interfacial aromatic molecules preferentially align their aromatic plane almost parallel to the hydrate surface, consistent with data reported in literature for aromatics and model asphaltenes at gas-water, oil-water and oil-solid interfaces^39,53,54^. When AAs are present, monocyclic aromatics (benzene, toluene, p-xylene) shows isotropic orientation distributions. On the contrary, polycyclic aromatics, including naphthalene and pyrene, tend to align perpendicularly to the hydrate surface (Fig. 5d). These results are consistent with the monocyclic aromatics having the effect of disrupting the order of the AAs within the interfacial film, and polycyclic ones to induce order, at least at low AAs surface density.

### Ordering of AAs at hydrate/oil interface

To quantify synergistic or antagonistic effects, we monitored changes in the structure of the AAs within the interfacial film due to the presence of aromatics. It should be noted that the order of the interfacial film was found to be correlated with the AAs performance, as well as with the ability of methane to diffuse from the hydrocarbon phase to the hydrate substrate in our prior studies^8,27^. To quantify the order of the AAs films, we calculated the deuterium order parameter, S_CD_, for carbon atoms in the long alkyl tails of the AAs. To quantify S_CD_ with respect to the Z direction of the simulation box, we computed:

2 $${{\bf{S}}}_{{\bf{C}}{\bf{D}}}\,=\frac{3\langle {\bf{c}}{\bf{o}}{{\bf{s}}}^{2}{\boldsymbol{\varphi }}\rangle -1}{2}$$

**Figure 6:**
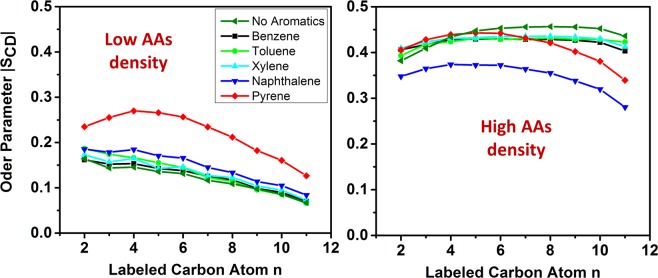
Deuterium order parameter for carbon atoms in the long tails of AAs in the presence of aromatic compounds. Results are obtained at AAs surface density of 0.44 (left) and 0.89 molecules/nm^2^ (right).

In Eq. (2), $\varphi$ is the angle between the C–H bond of carbon *i* in the alkyl tails and the Z direction of the simulation box. It is worth pointing out that $|{{\bf{S}}}_{{\bf{C}}{\bf{D}}}|$ = 0.5 indicates an alkyl tail perfectly ordered in *all-trans* conformation. The results, presented in Fig. 6, were obtained for the systems at two AAs surface densities: 0.44 and 0.89 molecules/nm^2^, respectively.

At low AAs surface density, the results (left panel of Fig. 6) indicate that the presence of aromatic compounds enhances AAs ordering. The effect decreases in the order pyrene > naphthalene > xylene ≈ toluene ≈ benzene.

At high AAs density (right panel of Fig. 6) the interfacial films become well-ordered because of the interactions between the AAs long tails and n-dodecane^8^. The monocyclic aromatics seem not to affect much the ordering of the AA alkyl tails. This is likely because these aromatics are excluded from the interfacial films, as discussed above. However, when polycyclic aromatics are present, different effects were observed. The order parameter obtained in the presence of naphthalene is the lowest, because the relatively large naphthalene molecules reside in the interfacial films and disrupt its order. On the other hand, pyrene adsorbed in the interfacial films slightly reduces the ordering of carbon atoms at the end of the alkyl tails, far from the head groups. This is because pyrene molecules stack in an ordered fashion inside the interfacial films. However, because their molecular size (~ 0.9 nm) is not comparable with the length of the AA alkyl tails (~1.5 nm in *all-trans* conformation), they can only induce order within the region of the film closer to the hydrate.

### Effective hydrate-hydrate interactions

To stabilize dispersions, the surfactants should yield effective repulsions between two particles^55–57^. To quantify the effective hydrate-hydrate interactions in the presence of aromatics, AAs, and both aromatics and AAs, we estimated free energy profiles as two hydrate substrates approach each other along the Z direction of the simulation box.

**Figure 7:**
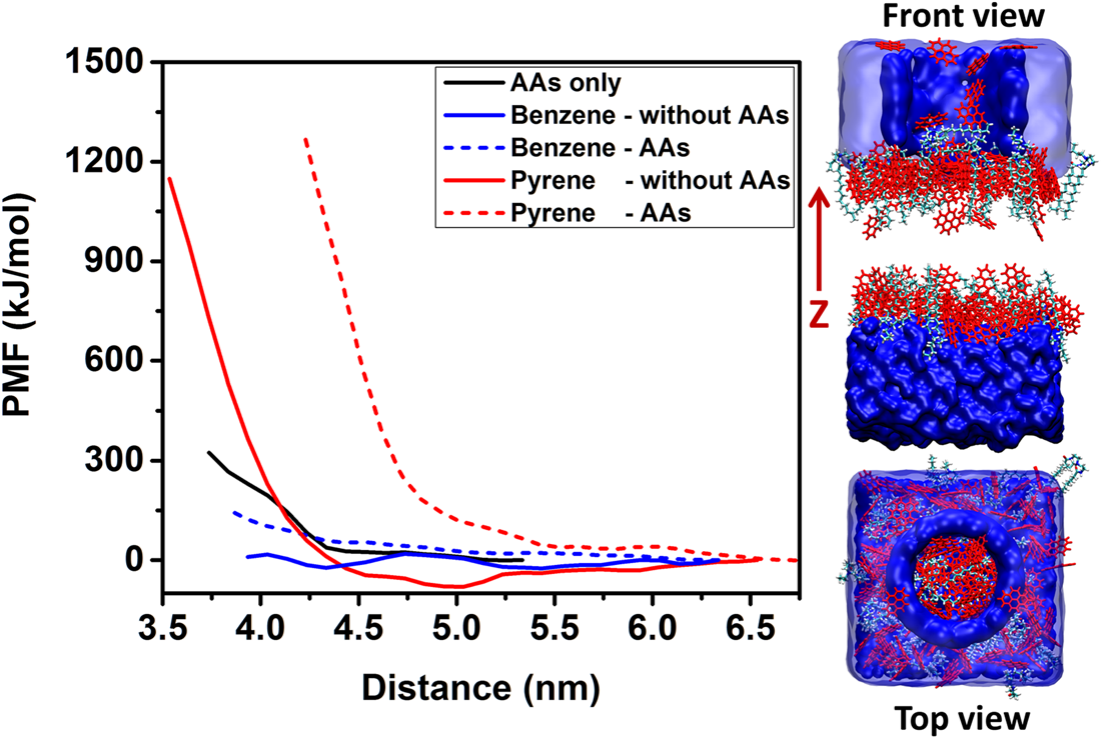
Left: potential of mean force calculated for systems in the presence of AAs only (black solid line); benzene and no AAs (blue solid line); benzene and AAs (blue dash line); pyrene and no AAs (red solid line); pyrene and AAs (red dash line). Right: a representative simulation snapshot showing water = blue, AAs = cyan, pyrene = red. Dodecane and methane are not shown for clarity.

To construct the initial configuration, we extracted one hydrate substrate with adsorbed AAs, aromatics, n-dodecane, and methane from the equilibrated systems. The substrate was duplicated. The two replicas were inserted facing each other in the same simulation box and separated along the Z direction. One channel of diameter ~3 nm was carved out of one of the two substrates, which allows molecules to move out from the region between the two hydrates as the two substrates approach each other. The free space of the simulation box was filled with n-dodecane. A simulation snapshot is shown in Fig. 7. Simulations were conducted as the centers of mass (COM) of the two hydrate substrates were constrained at different distances for 15 ns. The force applied to constrain the substrates was collected and averaged during the last 5 ns of each simulation. The PMFs were reconstructed by integrating the force-distance profiles, following examples from the literature^39,58,59^.

The results are shown in Fig. 7. Benzene alone was found not to induce appreciable repulsions between the approaching hydrate substrates (blue solid line). Benzene was also found to lower the repulsion induced by AAs (blue dash line vs. black solid line), suggesting that the presence of benzene could compromise the performance of the AAs considered.

On the contrary, our results show that pyrene induces an effective repulsion between two approaching hydrate substrates even when no AA is present (red solid line). The results are consistent with the suppressed cohesive force between water droplet/hydrate particle and hydrate particle in the presence of asphaltene or resin extracted from crude oils (at least by 2 orders of magnitude compared to the system without asphaltene or resin), which has been suggested to be due to the adsorption of asphaltene and resins on the hydrate-oil interface^48^. A shallow free energy well was observed for this system at ~5 nm. This is probably due to the fact that pyrene molecules strongly adsorb on hydrates surfaces, where they can promote π–π stacking;^60^ when they accumulate in the gap between two hydrates, pyrene molecules can form a bridge that contributes to bringing together the two hydrates. When AAs are added to the system with pyrene (red dashed line), the effective hydrate-hydrate repulsion is much stronger and longer-ranged compared to that observed with only pyrene. Because pyrene molecules embed within AAs films, they do not yield mid-range effective attractions between hydrates (see Fig. 5). The results above indicate that for systems without, or at low surface density of AAs, polycyclic aromatics can enhance the repulsion between hydrates, consistent with the experimental data according to which crude oils containing higher amounts of large aromatic compounds can yield higher emulsion stability^61^. These results suggest that pyrene could perhaps function as a natural AA, and that it could contribute to enhance the performance of the AAs considered in this work.

It is worth noting that we did not calculate the PMF profiles for the systems at high concentration of AAs because the order of the interfacial film expected at those conditions is not preserved once the channel shown in Fig. 7, right panel, is drilled within the hydrate substrate. However, building on arguments that relate emulsions stability to interfacial mechanic barriers or the film strength as measured by interfacial elasticity^62,63^, ordered and rigid interfacial films should yield strong repulsion between the two approaching hydrates^8,27^.

Simulation snapshots in Fig. 3 and data in Fig. 6 show that naphthalene disturbs the AA thin film, while the presence of monocyclic aromatics and pyrene has no, or at most little effect on the order of the interfacial thin film either by being excluded from the interfaces (for monocyclic aromatics) or by enhancing the order of the alkyl AAs tails. Therefore, comparing the observations reported here for different aromatic compounds, it is expected that the free energy barrier induced by the presence of naphthalene at the interface will be lower compared than that observed in the presence of pyrene.

## Conclusions

In summary, using classic molecular dynamics simulations, we investigated how selected aromatic compounds could act as natural AAs for stabilizing hydrate particles dispersed in hydrocarbons, as well as how they could affect the performance of synthetic AAs. By analysing density distributions, preferential orientation distributions, and adsorption free energy profiles, we observed a strong segregation, driven by internal energetic effects, of aromatics at the hydrate-oil interface. It was found that polycyclic aromatics exhibit stronger adsorption compared to monocyclic ones. Consequently, the adsorbed layers of polycyclic aromatics induce an effective repulsion between hydrate particles, which suggest these compounds could act as natural AAs and emulsifiers. The simulations also reveal that, depending on their molecular size, aromatic compounds can have both synergistic and antagonistic effects with respect to AAs. For example, polycyclic aromatics such as pyrene can enhance the order of the AAs film; hence promote stronger hydrate-hydrate repulsions. The monocyclic aromatics considered here, in some cases, disrupt the AAs film at low AAs surface density. At high AAs surface densities, monocyclic aromatics are found not to have strong effects on the order of AAs films. Although limited to the conditions considered in our simulations the results presented could provide important evidence to explain the performance of asphaltenes, which contain a substantial proportion of highly aromatic molecules and their derivatives, in preventing gas hydrate agglomeration, and could also help the community further the fundamental understanding of the diverse mechanisms responsible for the stabilization of dispersions using surfactants.
